# Feasibility of using a mobile App to monitor and report COVID-19 related symptoms and people’s movements in Uganda

**DOI:** 10.1371/journal.pone.0260269

**Published:** 2021-11-19

**Authors:** Levicatus Mugenyi, Rebecca Namugabwe Nsubuga, Irene Wanyana, Winters Muttamba, Nazarius Mbona Tumwesigye, Saul Hannington Nsubuga

**Affiliations:** 1 Makerere University Lung Institute, Kampala, Uganda; 2 The AIDS Support Organization, Kampala, Uganda; 3 Makerere University School of Public Health, Kampala, Uganda; 4 Department of Mathematics, Makerere University, Kampala, Uganda; University of the Witwatersrand, SOUTH AFRICA

## Abstract

**Background:**

Feasibility of mobile Apps to monitor diseases has not been well documented particularly in developing countries. We developed and studied the feasibility of using a mobile App to collect daily data on COVID-19 symptoms and people’s movements.

**Methods:**

We used an open source software “KoBo Toolbox” to develop the App and installed it on low cost smart mobile phones. We named this App “*Wetaase*” (“*protect yourself*”). The App was tested on 30 selected households from 3 densely populated areas of Kampala, Uganda, and followed them for 3 months. One trained member per household captured the data in the App for each enrolled member and uploaded it to a virtual server on a daily basis. The App is embedded with an algorithm that flags participants who report fever and any other COVID-19 related symptom.

**Results:**

A total of 101 participants were enrolled; 61% female; median age 23 (interquartile range (IQR): 17–36) years. Usage of the App was 78% (95% confidence interval (CI): 77.0%–78.8%). It increased from 40% on day 1 to a peak of 81% on day 45 and then declined to 59% on day 90. Usage of the App did not significantly vary by site, sex or age. Only 57/6617 (0.86%) records included a report of at least one of the 17 listed COVID-19 related symptoms. The most reported symptom was flu/runny nose (21%) followed by sneezing (15%), with the rest ranging between 2% and 7%. Reports on movements away from home were 45% with 74% going to markets or shops. The participants liked the “*Wetaase*” App and recommended it for use as an alert system for COVID-19.

**Conclusion:**

Usage of the “*Wetaase*” App was high (78%) and it was similar across the three study sites, sex and age groups. Reporting of symptoms related to COVID-19 was low. Movements were mainly to markets and shops. Users reported that the App was easy to use and recommended its scale up. We recommend that this App be assessed at a large scale for feasibility, usability and acceptability as an additional tool for increasing alerts on COVID-19 in Uganda and similar settings.

## Introduction

Coronavirus disease also known as COVID-19 was first detected in Wuhan city, China in December 2019. More than a year into the pandemic, the infection has spread rapidly across the whole world with all continents now affected. SARS-COV-2, the causative agent represents a potentially fatal disease that is of great global public health concern [1]. By 14^th^ March 2021, according to WHO Situation Report, the global cumulative number of confirmed cases was 119,220,681 and 2,642,826 (2.2%) registered deaths [2]. From the same report, the African Region had 2,948,316 confirmed cases and 74,686 (2.5%) deaths. In Uganda, the first case was reported on 21^st^ March 2020 and by 14^th^ March 2021 there were 40,544 confirmed cases and 334 (0.8%) deaths. Symptomatic patients present with fever, cough, sore throat, runny nose, myalgia and fatigue [3, 4], however a group of patients termed asymptomatic do not present with any of the symptoms. The asymptomatic individuals play a role in the spread of COVID-19 as they have been found to be infectious [5–7]. Timely and effective control measures are vital to reduce the spread of COVID-19 [8].

In the fight against COVID-19, several preventive strategies including isolation of cases, contact tracing, quarantine and social distancing have been used across the world to control the spread of the disease [9]. The other measure found useful in controlling the spread of COVID-19 from patients to health workers in China is what has been termed as eagle-eyed observer [10]. Contact tracing and isolation have been found to be effective strategies for control of infectious diseases including COVID-19 [11].

Mobile phone-based applications have been deployed in several health care programs with good acceptability and improved data reporting [12, 13], and more recently used to predict COVID-19 potential in United Kingdom and United States [14, 15] as well as in developing countries [16].

Though mobile Apps have demonstrated the theoretical effectiveness in different disease settings, concerns exist on potential privacy implications. A study to assess acceptability of App-based contact tracing for COVID-19 across different countries using online surveys in France, Germany, Italy, the UK and the US found that concerns about cyber security and privacy, together with lack of trust in government were major barriers to adoption [17].

Like other countries, the Ugandan government imposed non pharmacological interventions including closure of schools, churches, markets, shops, cinemas and many other places that encourage crowding of people and directed people to stay at home. The Uganda ministry of health used contact tracing as one of the measures for identifying contacts of confirmed COVID-19 patients. However, it remained quite challenging to trace all contacts based on recalls by the patients or relatives amidst recall bias and possible reluctance [18]. Further, it had been documented that some people infected with COVID-19 did not show symptoms and as such were dangerous infection sources [19]. Therefore, documenting and reporting of daily symptoms and people’s movements using an electronic system such as a mobile App was deemed helpful in increasing the alerts that can lead to early detection of cases and knowledge of which areas to avoid [20].

We could not find a mobile App developed to monitor COVID-19 symptoms and people’s movements in our setting. Therefore, this study aimed at developing a mobile App and pilot it to assess its feasibility as a tool to increase alerts for COVID-19 infection in a Ugandan setting.

### Study aim

To develop the “*Wetaase*” App and pilot it to assess its feasibility in increasing alerts for COVID-19 infection in Uganda.

## Materials and methods

### Developing the “*Wetaase*” App

The “*Wetaase*” App is in form of a diary, enabling people to record their daily symptoms related to COVID-19 and movements. The App includes a few straight forward questions, including baseline characteristics of the users. Each day, users are required to capture data on symptoms related to COVID-19 (e.g., fever, cough, shortness of breath, sore throat, fatigue and runny nose, movements names of places visited and means of transport used to visit them) and contact with someone suspected or confirmed as case of COVID-19. An algorithm was embedded in the App to flag suspected COVID-19 cases basing on the following criteria: any report of fever with any other COVID-19 related symptom or any report of movement to risky locations or any report of contact with a suspected or confirmed case.

The App was developed in consultation with health workers and Ministry of Health officials engaged in the management of COVID-19. The App was developed using KoBo Toolbox which allows one to use the App on a mobile phone and a tablet computer. We preferred this platform because it is easy to use, has capability to automate reports and it is open source software, so its use is sustainable. The platform also allows one to capture data in the App without internet connection, and a user may connect to the internet only when uploading the data. The design and Architecture of the App is detailed in S1 Appendix. The App’s source code can be found at https://drive.google.com/drive/folders/1OGjzX78Etwu9m7w-5ewX1DGHIXivadmS?usp=sharing.

### Application of the “*Wetaase*” App

The *“Wetaase”* App was applied to a selected population around Kampala, the capital city of Uganda.

#### Study design and setting

This was a prospective cohort study with mixed methods of data collection and analysis. The study included household members from 30 households selected from densely populated areas of Bwaise, Katanga and Makerere Kivulu around Kampala. The participants were followed for a period of 3 months. Data on COVID-19 related symptoms, movements and contact with suspected or confirmed case were collected on a daily basis and captured in the App.

#### Study participants

We purposively selected 10 households from each of the three study areas. All consenting household members from the selected households were included in the study. These were followed up for 3 months (90 days) during the period August-October 2020. We excluded: 1) households occupied by a single person, 2) households where no adult (>18 years) owned a smart mobile phone, 3) households with no adult that had attained at least secondary education (limited ability to enter and upload daily data), and 4) households planning to shift from the study area within the following 3 months.

#### Recruitment of study participants and study procedures

Two research assistants were trained on protocol and ethical considerations including protective measures against COVID-19. The assistants were worked with local council chairpersons to identify eligible households. They sought permission and written informed consent from the household head before the household would be enrolled. Once the household head agreed to participate in the study, each household member was then consented/assented and those who provided written consent/assent were enrolled into the study. One adult member with ability to use a smart mobile phone was identified and thoroughly trained in using the App. The research assistants installed the App on the smart phone of the trained household member. This person was responsible for entering data on COVID-19 related symptoms and movements made by each household member (him/herself inclusive). All household members were asked to report daily, to this person, any COVID-19 related symptom namely flue/runny nose, sneezing, cough, fever, difficulty in breathing, chest pain, loss of smell/taste, joint pains, sore throat, muscle aches, skipping meals, body fatigue, abdominal pain, diarrhea, nausea/vomiting, confusion/disorientation, and hoarse voice; and all movements made in the day. Each participant was given a paper diary where he/she would document his/her daily data and would give the diary to the person responsible for data entry into the App to upload their data. Whenever possible, the household member was asked to give the name and contact details (telephone number and/or physical address) of the person they met within a distance of one arm for at least 15 minutes. During the first two weeks, the research assistants regularly visited participating households troubleshooting any challenges with the App. Incoming data were monitored to ensure all households were reporting on a daily basis. Households not reporting were approached to establish the reasons of not reporting and were guided as required.

#### Data collection

To capture quantitative data, we developed a tool and programed it to be administered on a mobile device using the App. The tool was pre-tested before roll out of the study. Using this tool, which was embedded in the App, we collected data on demographic characteristics of participants once at enrolment, and daily on symptoms, movements and contacts. Participants were asked to upload their data on a daily basis. We also collected qualitative data by conducting exit interviews through meetings where participants shared their views about the App. Qualitative data were collected on the following aspects; whether the study participants: liked the concept of using the App, liked the App, found the App easy to use, found the App robust, would recommend the App to others, would recommend the App to be rolled out, would be willing to pay for the cost of internet data, and would be willing to participate in another similar study.

#### Data management and quality assurance

At the programming stage of the App, we ensured that obvious data error sources were addressed to ensure quality data. A server was set-up to enable real time data download. Data were backed up in secure multiple locations to avoid data loss. The automated real time reports flagged people reporting COVID-19 related symptoms. The study coordinator monitored daily automated reports and informed the study doctor of any suspected cases (alerts). The study doctor assessed flagged participants to rule out other possibilities and thereafter referred those that needed to be tested to nearby COVID-19 testing centres for detailed screening and testing. Those who would have tested positive were to be managed by the Uganda ministry of health COVID-19 task force team.

#### Statistical analysis

Baseline participants’ characteristics were summarized using descriptive statistics e.g. proportions, means and median as appropriate. To determine the feasibility of the App, an indicator variable indicating which participants reported data on symptoms (if no symptom, one would be required to choose the “No” option), movements, and contacts on a daily basis was generated. Using this variable, a proportion of participants using the App on a daily basis was used to assess the feasibility of the App. A trend analysis of the daily proportion was performed to show how the usage of the App varied with time over the 90 days (3 months) of follow-up. Since data on the indicator variable were available on a daily basis, this resulted in longitudinal data. We used generalized linear mixed model (GLMM) to assess factors associated with the App’s usage while accounting for both individual and household level clustering. Factors with p-value less than 0.05 were considered statistically significant. Quantitative analyses were done using STATA version 14 software [21]. Qualitative data were analysed by presenting findings as verbatim quotations from the exit interviews. The results from qualitative data backed up the findings on feasibility from the quantitative analysis.

#### Ethics

All study participants were consented or assented before participating in this study. The study received ethical approvals from the Mengo Hospital Research Ethics Committee (Study number 33/5-2020) and the Uganda National Council of Science and Technology (Registration number HS742ES).

## Results

### Baseline characteristics

A total of 101 participants (Bwaise: 31, Katanga: 33, and Makerere-Kivulu: 37) were enrolled from 30 households. The median age was 23 (IQR: 17–36) years with 61% females. Table 1 shows the participants’ baseline socio-demographic characteristics distributed across the three study sites.

**Table 1:** Baseline socio-demographic characteristics of study participants by site.

Variable	Measure	Total	Bwaise	Katanga	Makerere-Kivulu
Participants	Number	101	31	33	37
**Number per household**	Median (IQR)	3 (3,5)	3 (2,5)	3 (3–5)	4 (3,4)
**Sex, n (%)**	Female	62 (61%)	22 (71%)	22 (67%)	18 (49%)
**Age of participant at enrolment (years), n (%)**	Median (IQR)	23 (17–36)	20 (18–33)	27 (12–36)	24 (18–40)
	<12	18 (18%)	3 (10%)	8 (24%)	7 (19%)
	12–17	9 (9%)	3 (10%)	4 (12%)	2 (5%)
	18–24	29 (29%)	15 (48%)	4 (12%)	10 (27%)
	35–34	17 (17%)	3 (10%)	7 (21%)	7 (19%)
	≥35	28 (28%)	7 (22%)	10 (30%)	11 (30%)
**Age of data entrant (years), n (%)**	18–24	34 (34%)	17 (55%)	2 (6%)	15 (41%)
	25–34	41 (40%)	6 (19%)	13 (39%)	22 (59%)
	> = 35	26 (26%)	8 (26%)	18 (55%)	-
**Ever attended school, n (%)**	Yes	87 (86%)	28 (90%)	30 (91%)	29 (78%)
	No	10 (10%)	2 (6%)	2 (6%)	6 (16%)
	Infant	4 (4%)	1 (3%)	1 (3%)	2 (5%)
**Highest level of Education, n (%)**	Pre-Primary	34 (39%)	10 (36%)	14 (47%)	10 (34%)
	Primary	37 (43%)	14 (50%)	11 (37%)	12 (41%)
	Post-Primary	16 (18%)	4 (14%)	5 (17%)	7 (24%)
**Marital status, n (%)**	Married	28 (28%)	8 (26%)	12 (36%)	8 (22%)
	Not married	47 (47%)	17 (55%)	11 (33%)	19 (51%)
	Under age/students	26 (26%)	6 (19%)	10 (30%)	10 (27%)
**Occupation n (%)**	None/House wife	14 (14%)	5 (16%)	5 (15%)	4 (11%)
	Informal	39 (39%)	12 (39%)	12 (36%)	15 (41%)
	Formal	9 (9%)	-	6 (18%)	3 (8%)
	Under age	39 (39%)	14 (45%)	10 (30%)	15 (41%)

### The *“Wetaase”* App usage

Over the 90-day follow-up period of the 101 participants, we expected a total of 8,494 reports from the App; after discounting the withdrawals. However, we realized a total of 6,617 (78%) records; 1,800 from Bwaise, 2,330 from Katanga and 2,487 from Makerere-Kivulu.

### Reported symptoms

Each day, participants were required to use the “*Wetaase*” App to report COVID-19 related symptoms they may have experienced in the past 24 hours by selecting from the 17 symptoms. Out of the 6,617 records, 57 (0.86%) included a report of at least one COVID-19 related symptom. In total, 135 counts of symptoms were recorded; mostly (62%) from Bwaise. These are summarized in S2 Appendix. The most reported symptom was flu/runny nose at 21% followed by sneezing at 15%. The rest had rates ranging between 2% and 7%.

### Reported movements

We asked the participants if they ever moved out of their homes each day and where they went. Out of the 6,617 records, 3,002 (45%) included reports on movements away from home and these were highest in Katanga (68%) and lowest in Bwaise (20%). Majority of participants moved to markets or shops; 79% in Makerere-Kivulu, 72% in Katanga, and 70% in Bwaise. Details are presented in S3 Appendix.

### Reported contacts

No participant reported to have a contact with suspected or confirmed case.

### Feasibility of the “*Wetaase*” App

The overall usage rate of the App was 77.9% (95% confidence interval (CI): 77.0%–78.8%). Starting with a 39.6% (95% CI: 30.4%–49.6%) rate on the first day to a peak of 80.8% (95% CI: 71.4%–87.7%) on day 45 and declining to 58.7% (95% CI: 48.2%–68.4%) on day 90. Table 2 shows the overall, monthly, and household level rates of using the App during the study period by site. The color coding: red-green indicates the magnitude of the usage rate; red indicating the lowest and dark green the highest rates. Generally, the usage of the App was lowest in the first month and highest in the second month.

**Table 2:** Distribution of usage rates for the “*Wetaase*” App across households followed-up from August-October 2020 across the three study sites.

	Bwaise					Katanga					Makerere-Kivulu				
HH #	Expected entries	Aug-Oct	Aug	Sep	Oct	Expected entries	Aug-Oct	Aug	Sep	Oct	Expected entries	Aug-Oct	Aug	Sep	Oct
**All**	**2,352**	**77%**	**67%**	**89%**	**75%**	**2,970**	**78%**	**70%**	**83%**	**83%**	**3,172**	**78%**	**72%**	**85%**	**78%**
1	180	90%	83%	98%	88%	270	83%	79%	86%	86%	180	51%	43%	72%	37%
2	219	97%	100%	93%	100%	450	61%	35%	66%	81%	270	88%	86%	97%	81%
3*	39	51%	51%			270	66%	47%	74%	77%	360	89%	84%	100%	83%
4	180	84%	82%	73%	97%	270	25%	37%	31%	7%	200	44%	29%	48%	58%
5	450	77%	64%	83%	83%	270	94%	92%	92%	98%	180	89%	73%	97%	97%
6*	24	50%	50%			540	96%	91%	97%	99%	272	94%	84%	100%	100%
7	540	87%	76%	98%	89%	270	95%	88%	97%	100%	360	42%	53%	48%	25%
8	270	60%	43%	92%	44%	270	82%	69%	93%	83%	180	86%	80%	92%	87%
9	180	48%	27%	77%	40%	180	94%	92%	98%	92%	360	69%	53%	81%	73%
10	270	65%	60%	82%	53%	180	94%	83%	100%	100%	810	96%	93%	96%	100%

*These households in Bwaise withdrew from the study in the first month of follow-up.

**Fig 1:**
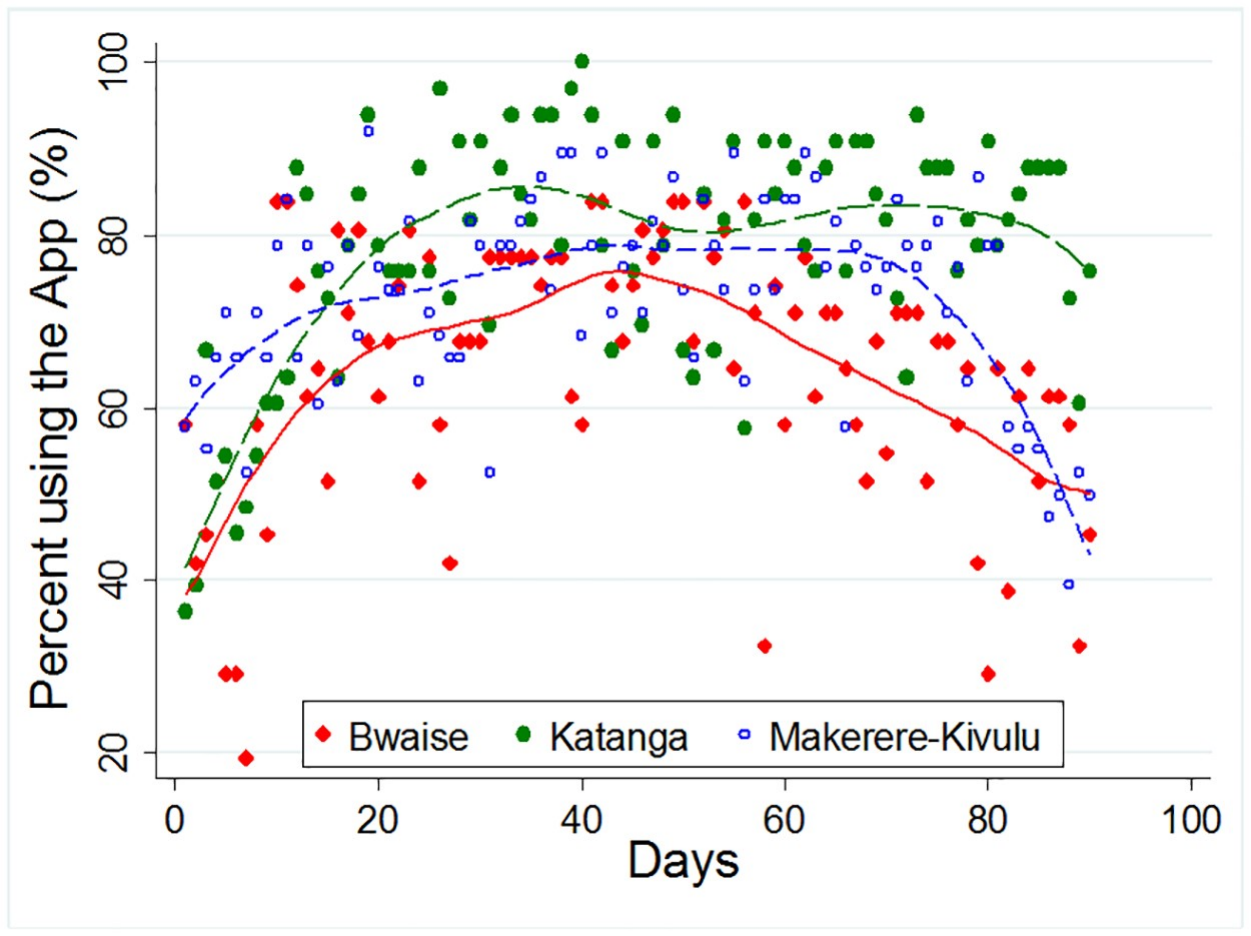
Average usability rates for the “*Wetaase*” App from day 1 to day 90 for the three study sites.

Fig 1 shows the trend of the average usability rate from day 1 to day 90 for the three study sites. Results indicate that the rates of using the App did not seem to differ by site, however, the peak was highest in Bwaise. While the App use declined during the third month, it remained high for Katanga compared to the other two sites. Similar results were found when stratified by sex and age groups.

Table 3 shows results from a univariate analysis using the GLMM model assessing how App usage varied by study site, sex, and age. The likelihood of using the App was higher in Katanga (odds ratio (OR): 2.68, 95% CI: 0.78–9.15, p = 0.12) and in Makerere-Kivulu (OR: 1.14, 95% CI: 0.37–3.50, p = 0.82) compared to Bwaise. However, these differences were not statistically significant, p value less than 0.05. Similarly, the use of the App did not differ by sex and age.

**Table 3:** Generalized linear mixed model estimates for usage of the “*Wetaase*” App.

Acceptability	OR	Std. Err.	z	P>|z|	[95% Conf. Interval]	
**Site (reference: Bwaise)**						
Katanga	2.68	1.68	1.57	0.12	0.78	9.15
Makerere-Kivulu	1.14	0.65	0.22	0.82	0.37	3.50
**Sex (reference: Male)**						
Female	0.85	0.10	-1.35	0.18	0.66	1.08
**Age (years) (reference: <12)**						
12–17	0.85	0.20	-0.71	0.48	0.54	1.34
18–24	1.22	0.24	1.03	0.30	0.83	1.80
>25+	1.32	0.22	1.68	0.09	0.96	1.84
**Data entrant age (years) (reference: 18–24)**						
25–34	0.71	0.39	-0.63	0.53	0.24	2.09
> = 35	0.42	0.29	-1.25	0.21	0.11	1.63

### Suspected cases and referrals

The 57 reports of COVID-19 related symptoms were investigated by the study doctor as soon as possible. The study doctor identified only 2 suspected cases of COVID-19. One of these was a female aged 18 years who reported all the 17 symptoms on the first and second day of follow-up. This participant reported to be asthmatic. The other participant was a male aged 53 years who reported fever, sneezing and muscle aches. The two suspected cases reported no history of travel outside Kampala or visiting a hospital in the previous 24 hours or getting in contact with a suspected case of COVID-19. These two were referred to nearby testing hubs for further investigations and testing. Both participants tested negative for COVID-19.

### Qualitative findings

To a large extent, the participants liked the “*Wetaase*” App. For example, the participants said the App was easy to use, helped them to monitor their symptoms and contacts, and acted as a link to the doctor enabling timely medical support. The participants proposed that the App be scaled-up for use in the whole country to improve on the COVID-19 alert system. They also proposed ways of improving the App as summarized in Table 4.

**Table 4:** Selected responses from participants during exit interviews.

Supportive feedback	Feedback suggesting improvement
The App is very user friendly and can be used by any ordinary Ugandan who can read. I liked the idea of including English and Luganda* in the App which enabled clear understanding of the questions	Data entry was dependent on only one participant within the household. This was challenging in times when the trained person was busy, sick or unavailable. I recommend training more than one person within a household as back up to ensure consistency in data entry
The App has been very useful for monitoring our symptoms and contacts and should be rolled out to the whole country	Sometimes the App would malfunction and would require a study staff to physically come over and troubleshoot. I recommend training people within the community to troubleshoot and repair the App without necessarily waiting for study staff
I appreciated the team for the quick follow up and support I got when I had to test for COVID-19	Some questions were allowing only a limited number of entries yet there would be multiple options, for example the number of movements made within the day
I was able to consult the study doctor via a phone call (which was provided in the App) on other illnesses not necessarily COVID-19 related and I was always getting support	I sometimes met so many people that capturing this information was challenging, for example at burials or social events like weddings
	I sometimes forgot to capture contact details and vehicle number plates

*Luganda is a language locally used by majority of the people in the study setting. It can easily be edited in the App to suit a different setting.

## Discussion

Our study has shown that the “*Wetaase*” App is feasible; as indicated by an average usage rate of 78% with similar rates across sites, sex and age groups. The overall rate increased to a peak in the second month after which it declined to about 59%. The decline in usage of the App during the third month could have been due to fatigue. There was a low reporting of symptoms related to COVID-19 and substantial reporting of movements out of the home; mainly to markets and shops. No one reported to have had a contact with a suspected or confirmed case.

The participants reported to have liked the “*Wetaase*” App saying that it was easy to use, it helped them to monitor their symptoms and contacts, and it linked them to the doctor enabling timely medical support. The other reasons supporting the feasibility of this App include the following: the App was developed using free open-source software, the participants were willing to use their mobile phones, the App can work on a simple smart phone, and recent government reports indicate that internet coverage and usage has greatly improved in the country.

The low rate of reporting symptoms and reporting no contact with any suspected or confirmed case, could be because the study was conducted at the time when COVID-19 was not widely spread in the study communities; for example, Uganda had reported only 11,341 cases during the study period: August to October 2020 [2]. Also, participants could have been reluctant to report truthfully due to fear of being quarantined which was the practice during the study period.

The high usage rate of the “*Wetaase*” App is consistent with results from a study conducted in Ghana that aimed at assessing feasibility, usability and acceptability of a mobile App for community-based maternal, neonatal and child care. The Ghana study showed that the App was acceptable and it improved data reporting [12]. Also the challenges about our App are similar to those reported by the Ghanaian study where their App was reported to have been hindered by software and device challenges [12].

The study has some limitations. First, the App was piloted during the period when there were few cases of COVID-19 in Uganda. Secondly, the App used one doctor to assess participants flagged as potential cases. However, during the period of a fully blown epidemic, using a single doctor may not be plausible. In this case, we recommend that the alert message in the App be revised to ask flagged participants to contact the Ministry of Health on a specified toll free line or to go to a nearby health facility for assessment, instead of being asked to contact a single doctor. Thirdly, the App requires internet connection to upload data which may be costly to the users. We recommend that the government, through the Ministry of Health, collaborates with telephone network companies to allow a free internet access to the “*Wetaase*” App.

## Conclusions

Overall, the “*Wetaase*” mobile App is feasible; with similar usage rates across study sites, sex and age. Usage of the App increased with time to a peak of 81% on day 45 after which it declined to 59% on day 90. Participants were able to use the “*Wetaase*” App and recommended its roll out to monitor COVID-19 infection. With the reduced risk of being quarantined, since government now encourages home-based care, we believe reporting of symptoms related to COVID-19 within the “*Wetaase*” App would increase; and thus enable quick detection of potential cases. We recommend that this App be assessed, at a large scale, for feasibility, usability and acceptability as an additional tool for increasing alerts on COVID-19 in Uganda and similar settings.

## Supporting information

S1 Data CSV file. (CSV)

S1 Appendix Design and Architecture of the “*Wetaase*” App. (DOCX)

S2 Appendix Distribution of COVID-19 related symptoms recorded in the *“Wetaase”* App. (DOCX)

S3 Appendix Distribution of reports of movements from home and places visited. (DOCX)
