# Supplementary material for: Feasibility of using a mobile App to monitor and report COVID-19 related symptoms and people’s movements in Uganda
Source: PLoS One. 2021 Nov 19;16(11):e0260269. doi: 10.1371/journal.pone.0260269 (PMC8604357; doi:10.1371/journal.pone.0260269)
Supplement: S1 Appendix — (DOCX) [file pone.0260269.s002.docx]

**S1 Appendix: Design and Architecture of the “*Wetaase*” App**

KoBo Toolbox, an open-source application was used to develop the “*Wetaase*” App. For this App, two major components of Kobo Toolbox were used, namely Kobo Collect and Kobo Toolbox Server. The application designer created the data collection forms using KoBo Collect and configured a server for access, store and manipulate data.

***Kobo Collect:*** Kobo collect is an Android Application that was installed on the participants’ mobile phone device and used for data collection. Kobo collect is efficient and effective in collecting data using simple questions without relying on a network connection. A wide span of data can be collected using Kobo collect which includes location (GPS) data, free text or multi choice responses, images and audios if required. The use of validation techniques such as constraints and form logic improve the ease and quality of data submitted by participants. The Kobo collect forms captured participants’ socio-demographics, contact and symptom information.

Kobo collect was set up on mobile devices either manually or through Google Play store and saved on the device as an Android Package file (APK). The Application was configured to connect to a server from which users can download, fill, edit and submit completed forms.

***Kobo Toolbox Server:*** This is a web-based server used for storing and analysis of data collected using Kobo Collect forms. It operates well with different hosting environments and supports various data types. Using Kobo Toolbox server, blank study forms are hosted and collected data is stored. Real time insights into collected data is available through visualization using maps and graphs. Collected data can be exported into multiple formats for advanced data analysis.

***Data security and protection:*** To ensure data security and protection, authentication using password and username was required to download and fill forms, participants were not able to edit forms once submitted. All study personnel including the PI, co-PIs and data manager were required authentication to manage data and access the server. We used Secure Socket layer (SSL) certificates for server deployment. Frequent and routine data backups were done to protect against data losses.

***Use of GeoPoint data for tracking patients and contacts:*** Participants were requested to capture GPS data when submitting their information. This enabled access to GeoPoint data (Latitude and longitude points) making it possible to track and follow up suspected patients or contacts and to map and visualize clusters in the event that a COVID 19 case was diagnosed for faster tracing.

***Architecture Construct:*** The diagram below shows the system design described above.
