# Supplementary material for: Feasibility of using a mobile App to monitor and report COVID-19 related symptoms and people’s movements in Uganda
Source: PLoS One. 2021 Nov 19;16(11):e0260269. doi: 10.1371/journal.pone.0260269 (PMC8604357; doi:10.1371/journal.pone.0260269)
Supplement: S2 Appendix — (DOCX) [file pone.0260269.s003.docx]

**S2 Appendix: Distribution of COVID-19 related symptoms recorded in the *“Wetaase”* App**

| **Symptom** | **Number of symptom reports by site** | | | |
| --- | --- | --- | --- | --- |
|  | **Bwaise** | **Katanga** | **Makerere-Kivulu** | **Total  (% overall)** |
| **Total Symptom reports** | **84** | **34** | **17** | **135** |
| Flu runny nose | 12 | 10 | 6 | 28 (21%) |
| Sneezing | 12 | 6 | 2 | 20 (15%) |
| Muscle aches | 6 | 3 | 1 | 10 (7%) |
| Skipping meals | 2 | 7 | 1 | 10 (7%) |
| Body fatigue | 8 | 0 | 1 | 9 (7%) |
| Abdominal pain | 8 | 1 | 0 | 9 (7%) |
| Loss of smell or taste | 4 | 3 | 1 | 8 (6%) |
| Cough | 4 | 1 | 2 | 7 (5%) |
| Joint pains | 5 | 0 | 2 | 7 (5%) |
| Fever | 3 | 2 | 0 | 5 (4%) |
| Sore throat | 3 | 0 | 1 | 4 (3%) |
| Diarrhea | 4 | 0 | 0 | 4 (3%) |
| Difficulty in breathing | 3 | 0 | 0 | 3 (2%) |
| Chest pain | 3 | 0 | 0 | 3 (2%) |
| Nausea and vomiting | 3 | 0 | 0 | 3 (2%) |
| Confusion/disorientation | 2 | 1 | 0 | 3 (2%) |
| Hoarse voice | 2 | 0 | 0 | 2 (1%) |
