# Supplementary material for: Feasibility of using a mobile App to monitor and report COVID-19 related symptoms and people’s movements in Uganda
Source: PLoS One. 2021 Nov 19;16(11):e0260269. doi: 10.1371/journal.pone.0260269 (PMC8604357; doi:10.1371/journal.pone.0260269)
Supplement: S3 Appendix — (DOCX) [file pone.0260269.s004.docx]

**S3 Appendix: Distribution of reports of movements from home and places visited**

| **Site** | **Reports** | **Left home n (%)** | **Places visited (percentage of those that left home)** | | | |
| --- | --- | --- | --- | --- | --- | --- |
|  |  |  | **Market/Shop** | **City/park** | **Health Facility** | **Other** |
| Bwaise | 1,800 | 355 (20%) | 69.6% | 2.0% | 2.5% | 25.9% |
| Katanga | 2,330 | 1,585 (68%) | 72.0% | 4.0% | 1.1% | 22.9% |
| Makerere-Kivulu | 2,487 | 1,062 (43%) | 78.7% | 10.0% | 1.0% | 10.3% |
| **Total** | **6,617** | **3,002 (45%)** | **74.1%** | **5.9%** | **1.3%** | **18.8%** |
